# Annual performance evaluation of a hybrid concentrated solar–micro gas turbine based on off-design simulation

**DOI:** 10.1016/j.heliyon.2024.e30717

**Published:** 2024-05-03

**Authors:** SeyedVahid Hosseini, Yong Chen, Seyed Hossein Madani, Mahmoud Chizari

**Affiliations:** a University of Hertfordshire, Hatfield, AL10 9AB, UK; b NEX Power Ltd., Milton Keynes, MK11 3JB, UK

**Keywords:** Micro gas turbine, Hybrid, Solar dish, Off-design, Power generation

## Abstract

As the adoption of solar hybrid systems continues to rise due to their potential to compensate for the fluctuation of solar irradiation, it becomes imperative to accurately evaluate their performance, considering the variation of off-design conditions. This paper introduces a detailed analysis method for evaluating the annual performance of a solar-MGT system under transient boundary conditions for a whole-year operation range. A hybrid system of a micro gas turbine, recuperator, and solar dish is considered, and an off-design simulation model is developed and verified with available experimental results. Two different configurations for a recuperated cycle are considered, and simulations are conducted for a test case in Pretoria, SA. The results for Jun.21 and Dec.21 as low and high solar energy days are reported with more details to compare the configurations and demonstrate the effect of ambient temperature on the heat loss of the solar receiver and the overall performance of the system. The alternative configuration reduces heat loss with a lower temperature receiver but has higher fuel consumption compared to the conventional configuration. Operating strategies for different hours of operation from 1 h to 24 h per day are simulated for 365 days, based on real meteorological data, and compared with the operating in solar available hours. It is shown that the whole-year simulation of the system considering the variation of boundary conditions can change the estimation of fuel consumption by 25 %.

**Table:** Nomenclature

A	Area
C	Heat capacity rate
c_p_	Specific heat at constant pressure
CR_g_	Geometrical concentration ratio
D_h_	Hydraulic diameter
h	Enthalpy
K	Pressure-loss factor
L	Length
$\dot{m}$	Mass flow rate
N	Shaft rotational speed
p	Pressure
Q	Thermal energy transfer
$\dot{Q}$	Thermal energy transfer rate
R	Gas constant
T	Temperature
U	Overall heat transfer coefficient
V	Volume
W	Mechanical/electrical energy
$\dot{W}$	Mechanical/electrical energy rate
Greek letters	
η	Efficiency
α	Absorptivity
σ	Stefan–Boltzmann constant
Δ	Changes in
μ	Viscosity
ρ	Density
ϵ	Effectiveness
Ω	Combustion loading factor
Β	Auxiliary coordinate, compressor map
π	Pressure ratio
Subscripts	
in	Condition at inlet
out	Condition and exit
Dish	Related to Solar dish collector
Receiver	Related to Solar receiver
Solar	Related to Solar energy
radiation	Radiation heat transfer
convection	Convention heat transfer
Ambient	Ambient condition
0	Reference point
h	Heat exchanger's hot path
c	Heat exchanger's cold path
r	Ratio
min	Minimum value
max	Maximum value
ref	Reference location
d	Design point
cc	Combustion chamber
Abbreviation	
BC	Boundary Condition
UKRI	UK Research and Innovation
MGT	Micro Gas Turbine
DNI	Direct Normal Irradiation
NTU	Number of Transfer Units
CHP	Combined Heat and Power
TIT	Turbine Inlet Temperature
slpm	Standard litre per minute

## 1 Introduction

Solar power, being the most abundant renewable source of energy, has significantly contributed to the ongoing energy transition [1] with the capability to lead to future sustainable energy units in combination with other efficient generation systems [2]. Solar energy, specifically solar thermal utilisation, has attracted increased interest in recent years [3], and capacity of Concentrated Solar Power (CSP) has rapidly expanded in recent years, reaching 6430 MWe by December 2019 [4].

In CSP power generation technologies, solar irradiation is transformed into high-temperature thermal energy through the utilisation of reflectors (usually mirrors) that concentrate sunlight onto a line or focal point. These systems offer the potential to assist the total electricity system to link demand and supply and improve security of supply, and cost reduction potential through optimised identification of usage opportunities [5]. Solar concentrators offer flexible configurations that can be integrated with thermal engines for a variety of applications, including power generation, solar cooling/heating, and desalination, enabling the production of useful heat at different temperature levels ([2,6]).

The intermittent nature of solar resources, however, necessitates either storage system or hybridisation to secure power generation in response to varying demand. Some solutions have focused on the optimum utilisation of thermal energy through different ways of storage systems to use it at night or under unfavourable weather ([7,8]), considering high initial investments of large capacity heat storage. In the other approach, hybridisation can be achieved by integrating CSP systems with conventional power generators or by adding backup fuel, whether renewable like biogas and biodiesel, or fossil based. The adoption of the solar hybrid concept presents a promising solution to mitigate the dependence on fossil fuel energy sources while also harnessing the abundant renewable solar energy available [9]. In this way, solar irradiation can heat a heat transfer fluid at the receiver and takes part in a thermal power engine to generate electricity. Among other thermal engines, a micro gas turbine (MGT) has the flexibility to combine with other sources to attain an optimal hybrid system [10]. This combination operates in the Brayton cycle, which is reported to be the 3rd generation of the CSP technology [4] at higher temperatures (more than 700 °C) where valuable advanced outputs (e.g. electricity and fuels) can be produced [2].

Among various solar collector types, the heliostat field with central tower and Parabolic Dish Concentrator (PDC) demonstrate being capable of elevating the temperature of the working fluid to the above-mentioned level. These systems have recently attracted more attention as a reliable ways to harness solar power in the form of electricity and increasing the power density in microgrids, while solar towers are better suited for higher power ranges [11]. PDC has the highest optical efficiencies and the highest concentration ratios [12], which in combination with high receiver temperature, can attain the highest ideal efficiency of the system [13].

Moreover, MGT present a promising technology for small-scale and distributed power generation systems, benefiting of low vibration and noise levels, low operational costs, high fuel flexibility, and low emissions [14], which aligns well with the energy roadmap to 2050 for on-site, small-scale power generation, combined with heat recovery from the exhaust. Notably, their fuel flexibility enables switching from fossil fuels to carbon-free alternatives like ammonia and hydrogen [15].

**Fig. 1:**
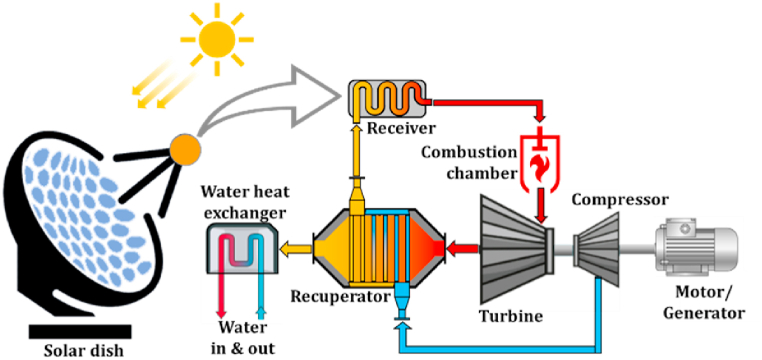
Schematic arrangement of the integrated system of a parabolic solar dish and micro gas turbine equipped with thermal energy storage and recuperator.

The system of a parabolic solar receiver (solar dish) with a micro gas turbine is shown schematically in Fig. 1. The solar irradiation captured by the dish is reflected by the solar receiver in its focal point, which could be equipped with thermal energy storage to compensate for the irradiation fluctuations [4]. The thermal energy is utilised to increase the temperature of the compressor outlet pressurised air. This high-temperature air can be heated up in the combustion chamber, which subsequently powers the turbine. MGT systems in the power output range of up to 20 kW are typically characterised by low-pressure ratios in the range of 2–3 [16]. Recuperation is normally incorporated to improve the cycle thermal efficiencies.

The first noticeable attempts to integrate the Brayton cycle with the CSP system were conducted by NASA and internal contractors in the 1980s, in which they investigated and demonstrated the concept and components in 5–85 kW systems ([[17], [18], [19]]) and in MW scales [20]. As a result, commercial CSP plants gained considerable momentum between 1984 and 1995 [21]. The micro capacity CSP-MGT systems have been investigated and demonstrated through several European projects after 2000. The overall objective of these projects, which are summarised in Table 1, was to showcase the technical feasibility and potential cost reduction of solar-hybrid systems, focusing on enhancement and optimisation of component design and performance ([[22], [23], [24]]).

**Table 1:** Recent demonstrated CSP-MGT project.

Project	Duration	Specification	Capacity	Reference
SOLGATE	2001–2003	Solar Tower/MGT	250 kWe	[23]
SOLHYCO	2006–2010	Solar Tower/MGT	100 kWe	[24]
OMSoP	2013–2017	Solar dish/MGT	3-10 kWe	[22]

The project “SOLGATE” started in 2001 with the aim to demonstrate the technological feasibility and validate the potential for reducing electricity generation costs with such a hybrid solar plant. A solar-hybrid test system with the design power output of 250 kWe was developed based on the Allison 250 helicopter engine, which was modified to integrate with external solar heat. Integration, commissioning and tests were conducted in the solar tower test facility of the PSA (Plataforma Solar de Almería, Spain), during which the receiver outlet reached temperature of 800 °C and power output of 230 kWe was recorded. The feasibility of commercial power plants employing industrial gas turbines Heron H1, Solar Mercury 50 and Nuovo Pignone PGT10 rating 1.4, 4.2 and 16 MW was also studied within this project [23].

Project “SOLHYCO” started in 2006 as a continuation to the “SOLGATE” to step forward commercialisation. The Turbec T100 Power and Heat unit (currently Ansaldo Energia AET100) was chosen to provide the capability to use bio-fuel, thus ensuring 100 % renewable operation. It was modified to integrate with the solar receiver of the CESA-1 tower at the PSA in a parallel combustion arrangement. Two hours of the smooth operation of solar-only micro-gas turbine operation was recorded unintendedly when a combustor flame-out went undetected by the control system. Despite various challenges in controlling parallel heat sources, 165 h of accumulated operation and maximum output power of 70 kWe was realised [24].

In 2013 a European project named “OMSoP” aimed to develop and demonstrate a small pure solar MGT system in the range of 3–10 kW. Experimental tests had the challenge of overheating in system bearings, constraining the increase of turbine inlet temperature (TIT) beyond 270 °C, which was signifanctly lower than the intended design point of 800 °C. As a result, the electricity output from the experimental tests was reported as low as 0.9 kW ([22,25]). These challenges consequntly abbreviated the testing phases of these projects.

Kasaeian et al. [2] published a review paper that explores the utilisation of various solar energy technologies whithin poly-generation system. They noted that the literature on solar-driven polygeneration systems appears to quite extensive, with a wide range of studies available. However, they concluded that there is a need for deeper investigation in the remaining domains; one of which is a need to establish a systematic methodology for investigating and the evaluating of the solar-driven systems to compare different designs. Prior research on hybrid solar MGT systems has primarily emphasised solar components, with comparatively less attention given to the gas turbine ([[26], [27], [28], [29]]). The conventional approach was to adapt an available gas turbine in the required power range with solar system [30]. Another approach involves developing a Solar MGT system based on off-the-shelf turbochargers, as studies by Gallup et al. [31], Kesseli et al. [32], le Roux et al. ([[33], [34], [35]]) and Ssebabi et al. [9]. Turbocharger components was employed to ensure cost-effectiveness and enhance the reliability of the turbomachinery but suffered from performance loss in the off-design condition of turbochargers.

Alzaili and Sayma [11] reviewed technical challenges related to using MGT for utilising concentrated solar power based on their experience from project OMSoP. Considering the economic aspects of the system, they estimated a cost breakdown of a pure solar MGT system at the rate of 5–10 kW and revealed that more than 60 % of the cost is for the dish and its accessories. It was concluded that careful consideration is needed in the design of the MGT generator to use the dish optimally and efficiently and to maximise the electricity production for a given dish through design optimisation.

Lanchi et al. [36] presented the simulation procedure based on components maps to analyse the behaviour of pure solar MGT of the project OMSoP in different meteorological conditions to define the optimal system parameters and control strategy. Based on their results, the annual average output power of a system with a nominal rate of 4.5 kW was 3.58 kW.

In a recent publication, Ghavami et al. [37] presented a methodology to optimise a pure solar MGT system considering technical, economic, and operational aspects. Their performance model calculated the off-design operation of the solar-MGT system by generating and scaling components maps which did not deal with the geometry of components. Based on their results in Seville (Italy), the Levelized Cost of Electricity (LCOE) for pure solar dish-MGT decreases to 85 €/MWh at an annual production rate of 10,000 units per year, which sounds promising since it is in the same range as the average cost of electricity from PV in the same area. Furthermore, the economic viability of solar power plants is influenced by their real lifetime and performance. Despite manufacturers data, empirical evidence suggests that the actual lifetime of PV power plants [38] and their performance efficiency [39] falls short of expectations, particularly in regions with tropical climates. This discrepancy underscores the importance of robust and reliable energy generation systems, such as hybrid solar-MGT systems, which offer not only environmental benefits but also economic advantages through enhanced durability and performance.

The performance of a solar hybrid MGT is affected by both solar input and ambient conditions, and it is required to see how much fuel it needs to consume in various operation conditions [40]. However, the studies on thermodynamic performances and capacity optimisation of hybrid solar systems have not included off-design conditions, particularly by considering real weather condition and hourly power & heat load data [41]. While prior works on microturbine performance modelling have been conducted, there was a lack of systematic simulation of the off-design performance of an MGT in integration with renewable sources [15] and part-load studies are largely performed only for the commercial systems at ISO conditions [42].

For a precise estimation of the performance of a Solar-MGT system, hourly simulations subject to different climates is demanded. Ssebabi et al. [9] simulated the performance of a solar hybrid MGT in both steady-state and transient boundary conditions with a quasi-steady approach based on hourly ambient conditions. However, their modelling limited to four representative solar days, one for each of the climatic seasons experienced in South Africa.

Although extensive research has been conducted in the past, there is currently a lack of commercially available off-the-shelf solar-hybrid micro gas turbine systems. Various operational and control challenges persist, necessitating significant development efforts to produce technically validated units. In almost all of the past research, the flow configuration between the turbomachinery, recuperator, and receiver was assumed to be fixed. A limited number of studies have evaluated the annual performance and addressed the off-design condition of a solar-MGT system, while the off-design behaviour of the components and their effects on the annual performance of such systems is rarely ever addressed for a pure solar MGT ([34,35]) and few other solar hybrid systems ([3,36,37]). Particularly, the available literature does not consider the effect of ambient temperature on both the performance of the MGT components and heat loss in the solar receiver for a complete operating range of the system.

This paper addresses the existing research gaps by introducing a detailed design/off-design analysis method for evaluating the performance of a solar-MGT system integrated with a parabolic dish concentrator under transient boundary conditions for a whole year operation range. A modular modelling approach considering the off-design condition of each component is employed and verified with available experimental results. The effect of ambient temperature on MGT performance, receiver heat loss and solar efficiency is considered, which distinguishes the performance of the system at various times of the day. A test case application in Pretoria, South Africa, is determined, a whole year real meteorological data is used, and two different configurations for the recuperated cycle are built and compared with this simulation approach to evaluate the annual performance of the system.

This study partly contributed to a UKRI-funded project, “SolarTurbo-CHP”, to develop a semi-renewable, grid-independent micro combined heat and power system and to improve energy accessibility in remote locations [39]. The aim of this project was to develop an optimal system and operation and control strategies for such a system. The experimental results of this paper are extracted from the MGT development phase of this project in the UK and the demonstration of its solar MGT system in Pretoria, South Africa.

## 2 System configuration and components

**Fig. 2:**
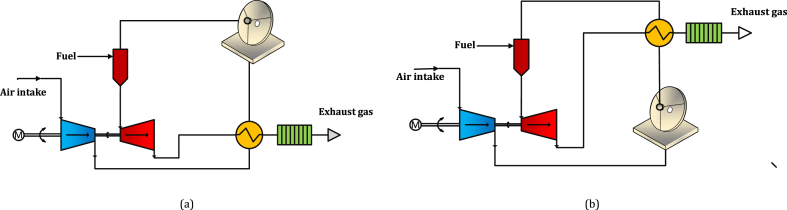
Configuration variations of a recuperated CPD-MGT system; (a) compressor flow recuperation, (b) receiver flow recuperation.

The Solar-MGT hybrid system consisted of the parabolic solar dish, receiver, micro gas turbine, combustion chamber and recuperator. The recuperator traditionally provides heat recovery between the compressor output and turbine output flow, as shown in Fig. 2(a). The other configuration considered in this research is shown in Fig. 2(b), where the recuperation is conducted between receiver output and turbine output flow. The former will be recalled as “Compressor flow recuperation” and the latter as “Receiver flow recuperation” through this paper.

## 3 Methodology and performance simulation model

The analysis is based on thermodynamic simulation of both the gas turbine components and solar part for each configuration to deliver a specific power output. The annual distribution of ambient temperature, pressure, humidity and DNI is considered the as boundary conditions (BCs). The quasi-steady approach, similar to Ref. [9], is employed to predict the system's performance due to the transient changes in boundary condition. The quasi-steady approach cannot capture dynamics of the system. Acknowledging that the dynamic response of the MGT system in much faster than the changes in boundary condition and resolution of this study, neglecting these dynamics does not affect the performance of the system in annual scale [[43], [44], [45], [46]].

The 0-D governing equations of the components are presented in a generic form. Gas turbine components and the overall matching algorithm are based on Walsh [47] but modified for each case which is explained in this section. The pressure and heat loss between the components are likewise modeled using a 0-D approach. The model is developed in MATLAB®. The system of equations, including mass, energy, and heat transfer, is solved using a multi-dimensional Newton-Raphson method.

### 3.1 Solar dish and receiver

The performance of the solar receiver in the cycle is referred to the solar energy that transfers to the working fluid ($Q_{Solar}$) and the pressure drop (Δp), which can define the temperature and pressure output of this component. The efficiency of the solar part of the system is related to the efficiency of the solar dish and the solar receiver.

The total incident solar heat on the solar dish can be calculated as equation (1):

1 $$Q_{in,Solar}=A_{Dish}DNI$$

where $A_{Dish}$ and DNI are the total area of the solar dish and Direct Normal Irradiance, respectively. The heat delivered from the dish and absorbed by the receiver ($Q_{in,Receiver}$) is calculated as equation (2) [48]:

2 $$Q_{in,Receiver}=Q_{in,Solar}\eta_{Dish}\alpha_{Receiver}$$

where $\alpha_{Receiver}$ and $\eta_{Dish}$ are the absorptivity of the receiver and the efficiency of the dish, respectively. A part of this energy is lost to the ambient by radiation and convection, and the rest of it is absorbed by the working fluid. So, the energy balance can be written as equation (3).

3 $$Q_{Solar}=Q_{in,Receiver}-Q_{loss,radiaion}-Q_{loss,convection}$$

For the sake of simplicity, the overall efficiency of the solar receiver system ($\eta_{Solar}$) is defined in equation (4) as the ratio of heat absorbed by the working fluid to the available heat at the dish area ($A_{Dish}$).

4 $$Q_{Solar}=\eta_{Solar}A_{Dish}DNI$$

This efficiency varies with the operating condition of the dish-receiver and can be obtained by integrating the above equations in the form of equation (5) to evaluate the overall solar performance in off-design conditions.

5 $$\eta_{Solar}=\eta_{Dish}\alpha_{Receiver}-\frac{\alpha_{Receiver}\sigma_{Receiver}\left({T_{Receiver}}^{4}-{T_{Sky}}^{4}\right)}{{CR}_{g}DNI}-\frac{U\left(T_{Receiver}-T_{Ambient}\right)}{{CR}_{g}DNI}$$

Receiver pressure loss depends on its geometry, mass flow rate and temperature. It has been shown by Shah and Sekulić [49] that the pressure loss in heat exchangers can be determind from equation (6), since the turbulent flow regime is dominant in the receiver.

6 $$\Delta p=0.023\frac{\mu^{0.2}}{\rho }\frac{4L}{D_{h}}\frac{{\dot{m}}^{1.8}}{{A_{0}}^{1.8}{D_{h}}^{0.2}}$$

### 3.2 Recuperator

The energy balance of the recuperator can be simulated by using effectiveness (ϵ), which defines as the ratio of the actual heat transfer from the hot to cold fluid, $Q_{Recuperator}$, to the maximum possible heat transfer. It can be rewritten as equation (7) to relate the enthalpy of the cold and hot flow paths.

7 $$\epsilon =\frac{h_{c,o}-h_{c,i}}{h_{h,i}-h_{c,i}}$$

The part-load performance of the recuperator and the relation between the effectiveness of a heat exchanger and the mass flow depends on its geometry [37]. It can be estimated, however, by using the well-known ϵ−NTU of counterflow-type heat exchangers [42] as presented in equation (8).

8 $$\epsilon =\frac{1-\exp\left[-NTU\left(1-C_{r}\right)\right]}{1-C_{r}\;\exp\left[-NTU\left(1-C_{r}\right)\right]}$$

The number of transfer units (NTU) and the heat capacity ratio ($C_{r}$) are defined by equations 9, 10, respectively [50].

9 $$NTU=\frac{UA}{C_{\min}}$$

10 $$C_{r}=\frac{C_{\min}}{C_{\max}};\;where\;C=\dot{m}c_{p}$$

### 3.3 Combustion

The combustion efficiency and pressure drop of the combustion are evaluated using the analytical equation suggested by Lefebvre [51]:

11 $$\frac{\Delta P}{P_{in}}=K\frac{R}{2}{\left(\frac{{\dot{m}}_{in}\sqrt{T_{in}}}{A_{ref}P_{in}}\right)}^{2}$$

12 $$\eta_{CC}=1-\left(1-\eta_{CC,des}\right)\frac{\Omega }{\Omega_{des}}\text{;}$$

$$where\;\Omega =\frac{\dot{m}}{{P_{in}}^{1.8}V_{ref}e^{T_{in}/300}}$$

Pressure drop can be rewritten as Equation (13) to ease the estimation of off-design condition:

13 $$\frac{\Delta P}{P_{in}}={{\left(\frac{\Delta P}{P_{in}}\right)}_{d}\times \left[\left(\frac{{\dot{m}}_{in}\sqrt{T_{in}}}{P_{in}}\right)/{\left(\frac{{\dot{m}}_{in}\sqrt{T_{in}}}{P_{in}}\right)}_{d}\right]}^{2}$$

In Equitation 11 to 13, $A_{ref}$ is reference area, Ω is the loading factor, and K is the pressure loss factor ($\frac{\Delta P}{q_{ref}}$) which is independent of the MGT working conditions. The constant values for the performance of the combustion chamber are extracted from an MGT combustion chamber designed by the authors [14].

### 3.4 Turbomachinery

The performance of compressors and turbines in off-design conditions is analysed using performance maps, which are constructed based on corrected parameters. The analysis of off-design conditions, then, is performed using quasi-dimensionless parameters: corrected mass flow rate ($\frac{\dot{m}\sqrt{T_{in}}}{P_{in}}$), corrected rotational speed ($\frac{N}{\sqrt{T_{in}}}$), pressure ratio (π), and efficiency (η) [45].

For the compressor, the quasi-dimensionless rotational speed and beta (*β*) serve as the basis for determining the quasi-dimensionless mass flow, pressure ratio (π), and isentropic efficiency (η), as written in equations 14, 15, 16, from the compressor performance maps. Here, *β* is an auxiliary coordinate that used to facilitate map interpolation [47].

14 $$\frac{{\dot{m}}_{in}\sqrt{T_{in}}}{P_{in}}=f_{1}\left(\frac{N}{\sqrt{T_{in}}},\beta \right)$$

15 $$\pi =\frac{P_{out}}{P_{in}}=f_{2}\left(\frac{N}{\sqrt{T_{in}}},\beta \right)$$

16 $$\eta =f_{3}\left(\frac{N}{\sqrt{T_{in}}},\beta \right)$$

In the turbine, the quasi-dimensionless mass flow and efficiency are treated as functions of the quasi-dimensionless rotational speed and pressure ratio as equations 17, 18.

17 $$\frac{{\dot{m}}_{in}\sqrt{T_{in}}}{P_{in}}=f_{4}\left(\frac{N}{\sqrt{T_{in}}},\pi \right)$$

18 $$\eta =f_{5}\left(\frac{N}{\sqrt{T_{in}}},\pi \right)$$

**Fig. 3:**
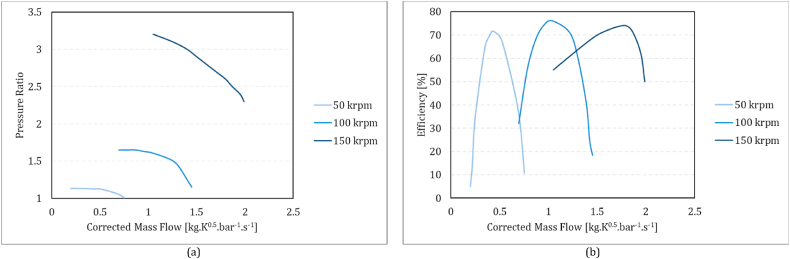
Compressor performance maps: (a) pressure ratio vs corrected mass flow rate and (b) isentropic efficiency vs corrected mass flow rate.

**Fig. 4:**
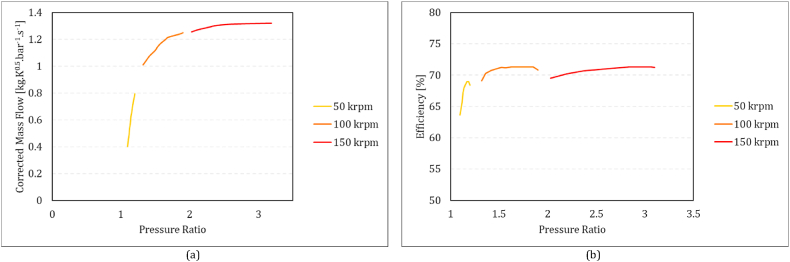
Turbine performance map; (a) corrected mass flow rate vs pressure ratio, (b) turbine efficiency vs pressure ratio.

Functions $f_{1}$ to $f_{5}$ can be portrayed as compressor and turbine characteristic maps. Conventionally, compressor maps show the pressure ratio and efficiency based on corrected mass flow rates in different corrected rotational speeds (Fig. 3 **a** and Fig. 3 **b**). For the turbines, these maps are the corrected mass flowrate and efficiency based on pressure ratio in different corrected rotational speeds (Fig. 4a and b). In this research, these maps adapted from the MGT prototype of the “Solar Turbo CHP” project.

## 4 Case study

The model is implemented to evaluate two different configurations for a micro solar dish power generation application in Pretoria, South Africa. South Africa is one of the leading countries in the deployment of solar dish power stations, with over 200 systems installed and a total capacity of 5 MW, according to the International Renewable Energy Agency (IREA). The South African government supports the growth of renewable energy, including solar, through initiatives such as the Renewable Energy Independent Power Producer Procurement Program, which aims to procure 11 GW of renewable energy by 2030. This program is expected to drive the growth of the solar energy sector in South Africa, including the deployment of solar dish power stations.

**Fig. 5:**
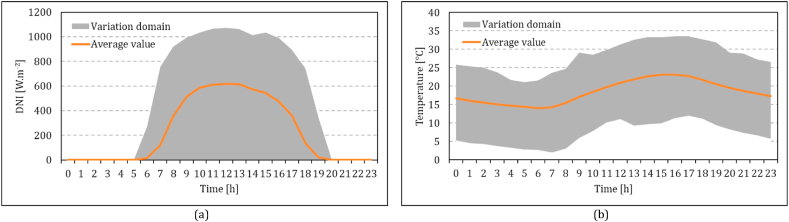
Variation of ambient condition in Pretoria, South Africa in 2022: (a) variation of DNI, (b) variation of ambient temperature; extracted from the Southern African Universities Radiometric Network database [52,53].

Environmental conditions, including temperature, pressure, relative humidity, and direct normal irradiance at Pretoria, are extracted from the Southern African Universities Radiometric Network database [52,53] for the weather station at the University of Pretoria. Variation of the DNI and ambient temperature values is shown in Fig. 5a and b, respectively.

## 5 Experimental validation

**Fig. 6:**
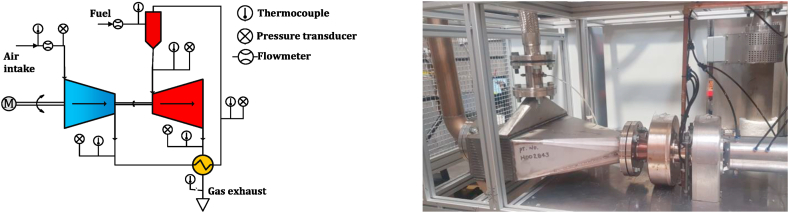
Recuperated micro gas turbine test cell and measurement positions.

The validation of the simulation model is conducted in two stages to ensure its accuracy and reliability. In the first stage, experimental data of the recuperated micro gas turbine on multiple full-load and part-load conditions is used to check the validity of the model for a wide range of operating points. In the second stage, limited experimental data from one of the configurations tested at the University of Pretoria, South Africa, is used to validate the model, including solar subsystems. These tests and experiments were conducted as part of the “Solar Turbo CHP” project. The test cell of the recuperated MGT as well as the schematic drawing showing the connections and measurement points, is presented in Fig. 6. A gas mass flow controller ensures precise measurement of the fuel. Also, a MAF sensor is used to monitor the compressor air mass flow rate. As illustrated in the schematic diagram of the test cell (Fig. 6), thermocouples and pressure sensors are installed at key locations, such as the compressor inlet and outlet, turbine inlet and exhaust, and other recuperator paths. A high-speed alternator was powered with a tie grid drive/inverter to enable a bi-directional connection to facilitate both motoring and generating and to monitor both the rotational speed and power output of the system.

**Fig. 7:**
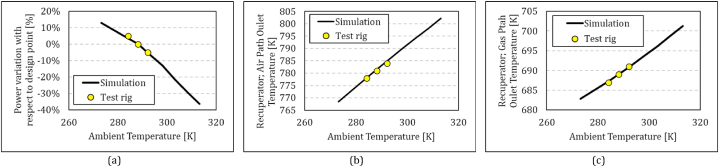
Comparison between simulations and experiments in off-design conditions; effect of ambient temperature on (a) power output, (b) outlet temperature of the recuperator in the air path, (c) outlet temperature of recuperator is gas path.

One aspect of the off-design simulation is to evaluate the performance in different ambient conditions. Fig. 7 demonstrates the output power variation (Fig. 7a), recuperator air path outlet temperature (Fig. 7b), and recuperator gas path outlet temperature (Fig. 7c) under different ambient conditions. The range of the experimental points is limited to the weather condition in the period of tests from 11° to 19 °C. The trends of the variations follow the well-known behaviours of gas turbines. It should be mentioned that the simulation model is tuned with regard to test results at 15 °C. The maximum error in power output is less than 3 % at 11 °C. The errors are higher in colder ambient temperatures due to higher heat loss of the system (especially the recuperator and its connections) in cold weather. This is also reflected in the turbine outlet temperatures, which show a difference of 4 °C. The recuperator heat loss yields to lower effectiveness values than expected and lower air outlet temperature consequently. A difference of less than 6 °C can be seen between the experiments and analysis.

**Fig. 8:**
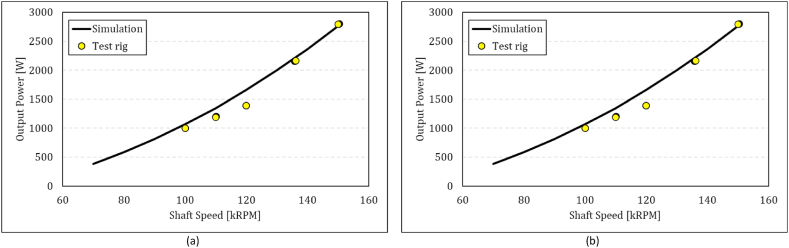
Comparison between simulations and experiments in off-design conditions: (a) power output and (b) fuel mass flow in various shaft speeds.

To demonstrate the validity of the model, the effect of shaft speed on the power output and fuel consumption is shown in Fig. 8a and b. Turbine inlet temperature is kept constant during this test. The trend of the variations is simulated properly, and the maximum error for the recorded data is 18 % and 20 % for the output power and fuel consumption, respectively.

**Fig. 9:**
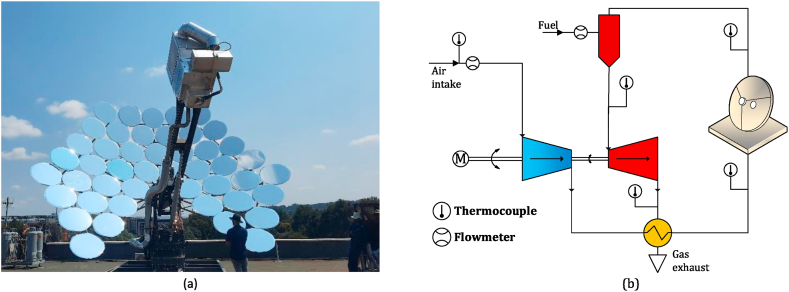
Solar MGT demonstration arrangement of the project “Solar Turbo CHP”, Pretoria, SA: (a) demonstration prototype, (b) schematic diagram of the system.

**Fig. 10:**
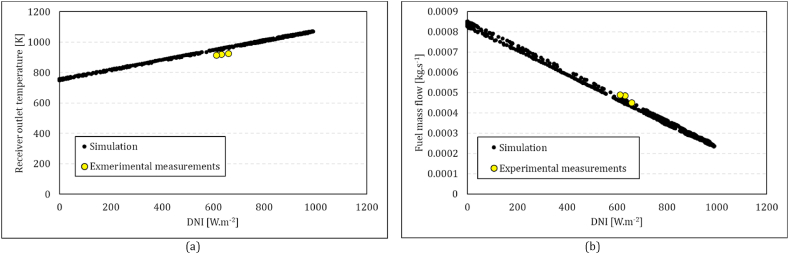
Comparison between simulations and experiments results of the solar MGT system: (a) air temperature at the outlet of the solar receiver, (b) fuel mass flow rate.

The solar dish MGT was integrated for the “Solar Turbo CHP” project in a conventional upstream recuperating configuration. The experimental demonstration setup and the schematic diagram are shown in Fig. 9a and b. The experimental results of the project were supplied by NEX Power Ltd, one of the project partners, and were used to validate the simulation platform in the Solar MGT arrangement. To verify the capability of the model in evaluation of the solar energy contribution, the air temperature at the outlet of the solar receiver (Fig. 10a) as well as the fuel mass flow (Fig. 10b), are compared with the experiments. The difference between the simulation and experiments for the temperature is less than 50 K (in the range of 1000 K), and the error in fuel mass flow is less than 5 % which validates the credibility of the model in a hybrid configuration.

## 6 Results and discussion

The results of the simulation study comparing the performance of two different Solar MGT configurations during a full year of operation are presented in this section. The performance of each configuration using various key performance metrics, including energy production, fuel consumption, solar contribution, and thermal efficiency, are analysed.

The off-design simulation model provides the capability to consider various control strategies. Here for the sake of simplicity in comparisons, constant TIT is selected for all the simulations and comparisons. Performance parameters of both configurations are first compared in more detail on some specific days to provide more resolution of simulation results, followed by a whole-year analysis. Then, a sensitivity analysis presents to identify the key parameters that impact the performance of the Solar MGT system, providing a better understanding of the factors that affect its operation.

**Fig. 11:**
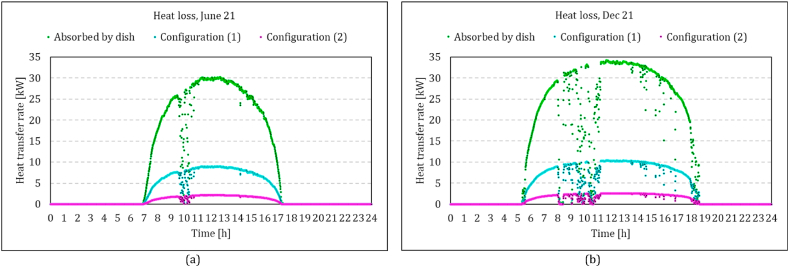
Comparison of the rate of main heat receiver heat loss between two configurations in both low and high DNI days of the year against the absorbed heat by the solar dish; Configuration (1): compressor flow recuperation, Configuration (2): receiver flow recuperation.

**Fig. 12:**
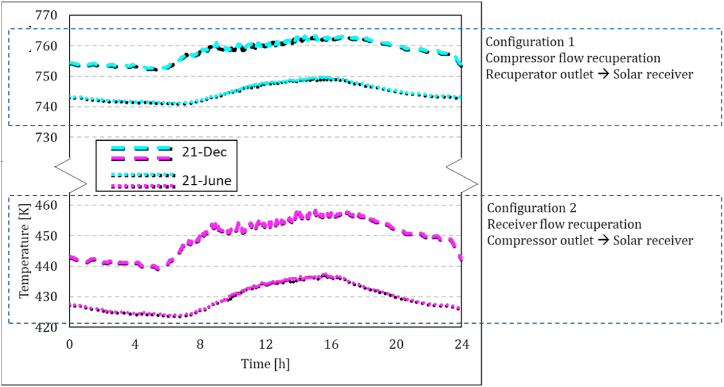
Solar receiver inlet temperature on 21-June and 21-December in both configurations.

Jun. 21 and Dec. 21 are considered representatives for a low and high DNI day, respectively. Fig. 11a-b presents a comparison of heat loss rates in two configurations, compressor flow recuperation (configuration 1) and receiver flow recuperation (configuration 2), in both low and high DNI days against the absorbed heat by the solar dish. It could be seen that the amount of heat loss is clearly proportional to the amount of the solar heat. The maximum value for configuration 1 is about 15 kW on Jun. 21 and 17 kW on Dec. 21. In contrast, the maximum value for configuration 2 is about 2–3 kW on Jun. 21 and Dec. 21. As was shown in equation (5), the efficiency of the solar receiver system is reduced due to the radiation and convection heat loss. The inlet temperature of the receiver in configurations 1 and 2 are recuperated air outlet and compressor outlet, respectively. Higher values of the receiver inlet temperature (Fig. 12) in Configuration 1 yield up to 5 times higher heat loss values, which means that in configuration 2, more solar heat is delivered to the working fluid.

**Fig. 13:**
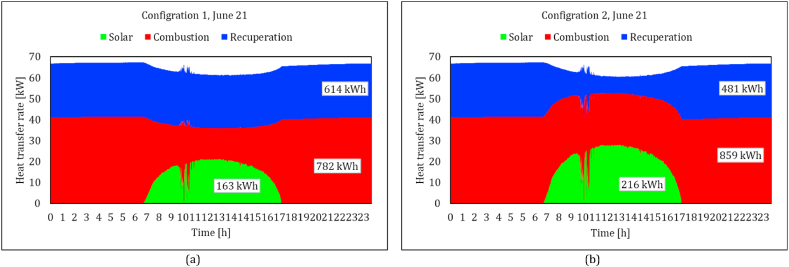
Comparison of the rate of main heat transfer mechanisms between two configurations in a low DNI day; variation of the solar heat which is absorbed by the working air, added heat by the combustion process and the recuperated heat; (a) compressor flow recuperation (b) receiver flow recuperation.

**Fig. 14:**
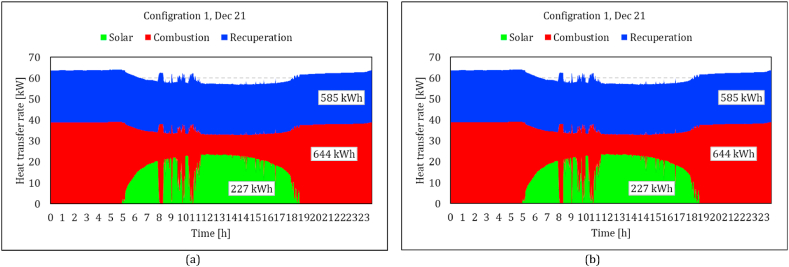
Comparison of the rate main heat transfer mechanisms between two configurations in a high DNI day; variation of the solar heat which is absorbed by the working air, added heat by the combustion process and the recuperated heat; (a) compressor flow recuperation (b) receiver flow recuperation.

To see the effect of solar heat on the energy balance of the system, the heat transfer rates in the solar receiver, combustion chamber and recuperator on Jun. 21 are shown in Fig. 13. Although the delivered solar heat in configuration 1 is considerably lower than configuration 2 (46 % less in the highest DNI), the effect of solar energy is higher in configuration 1. Combustion heat is reduced from 26.2 kW to 8.1 in Configuration 1 (Fig. 13a), while it reaches a minimum of 20 kW in Configuration 2 (Fig. 13b). The analysis is also processed for Dec. 21, which represents similar trends (Fig. 14a-b). Increasing the DNI during the day, the combustion heat reduced from 25 to 5 in Configuration 1 and to 17.8 in Configuration 2. It could be seen in both figures that the amount of recovered heat in the recuperator is significantly affected in Configuration 2 by increasing the solar heat. In other words, more solar heat is delivered to the working fluid in Configuration 2, but most of it substitute the recovery heat of the recuperator and does not affect the combustion chamber fuel consumption. The amount of recovery heat of the recuperator in Configuration 2, reduced from 25.7 kW to 7.7 kW (70 % decrease) on Jun. 21 and from 24.9 kW to 3.8 kW (85 % decrease) on Dec. 21, while it shows less than a 5 % decrease in Configuration 1.

The total consumed energy in both configurations seemed to be decreased by increasing the solar power. However, the reason is the higher ambient temperature in those high DNI periods. This effect could also be observed in the compressor outlet temperature (Fig. 12). Since the control strategy is assigned to keep the TIT constant, both the consumed energy and generated power are decreased. It is also worth mentioning that these two figures demonstrate that the model estimated more required combustion heat in the cold climate of Jun. 21 in no solar heat conditions. The total combustion energy is, however, less in Dec. 21 due to the wider window of available DNI. It indicates the importance of the simulation that covers all operating periods of the system.

**Fig. 15:**
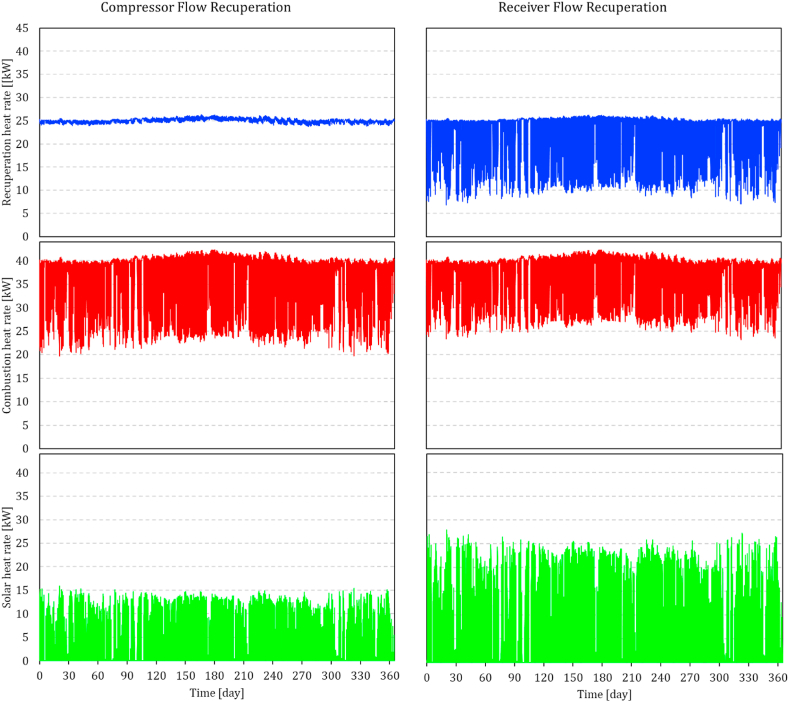
Heat transfer rate all over the year in both configurations: left compressor flow recuperation, right: receiver flow recuperation.

The simulation was conducted throughout the entirety of 2022. The results for the two configurations are plotted in Fig. 15. Indeed, it can be seen that the variation of solar irradiance does not have a considerable impact on the compressor flow recuperation configuration. As it was discussed in Fig. 11, Configuration 1 exhibits greater receiver heat loss compared to Configuration 2. Fig. 15 shows that the solar heat rate remains consistently below 16 kW throughout the year for configuration 1, whereas it peaks at nearly 28 kW for configuration 2. This higher solar heat, however, impacts the heat transfer in the recuperator. In configuration 1, it remains consistently around 25 kW, whereas in configuration 2, it occasionally drops below 10 kW. The maximum fuel consumption is nearly identical in both configurations, as there are numerous instances where the entire load is met by fuel. However, Configuration 1 exhibits frequent fuel consumption within the 20–25 kW range, an occurrence rarely observed in the other configuration.

The peak direct normal irradiance (DNI) occurs during the hottest times of the day, coinciding with reduced performance of the micro gas turbine (MGT). Consequently, variations in boundary conditions (BCs) can significantly influence the off-design performance of the hybrid system. To illustrate this effect, fuel consumption estimates using different methods are presented in Table 2. Three sets of assumptions were employed for these calculations. In the simplest method, the system is analysed based on annual average values of DNI, ambient temperature, pressure, humidity, and component design characteristics. In the second method, deviations from ISO conditions are considered, and estimates are based on off-design simulation. The third method, used in this study, incorporates minute-averaged values of DNI and ambient conditions alongside off-design simulation. The simplest evaluation results in a 21 % and 26 % overestimation compared to the comprehensive simulation conducted in this study for configurations 1 and 2, respectively. These deviations are 7 % and 1 % overestimates in the second approach.

**Table 2:** Annual fuel consumption (kg of propane) estimation with different methods.

Assumption	Configuration 1	Configuration 2
Average DNI and ambient conditions, components design data	29,215	30,879
Average DNI and ambient conditions, off-design simulation	22,346	24,131
Annual evaluation, off-design simulation (this study)	23,875	24,464

**Fig. 16:**
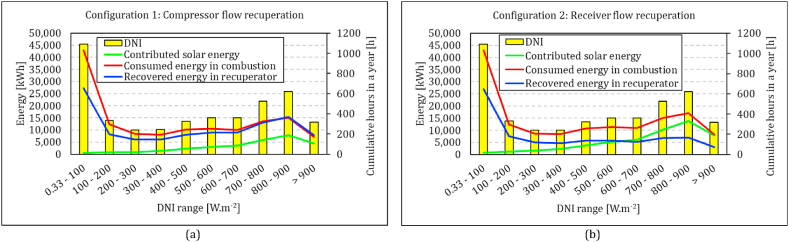
Heat transfer rate all over the year in both configurations: left compressor flow recuperation, right: receiver flow recuperation.

The proportion of each energy source in the system's energy balance varies across different scenarios. The primary parameter is the availability of solar energy, specifically the direct normal irradiation. In Fig. 16, the frequency distribution of different DNI levels is plotted alongside the contribution of each energy conversion within each level. The simulation covers continues operation for 24 h a day, 365 days a year. The first group comprises the DNI values below 100 W/m^2^, indicating occasions when solar availability is minimal. During these periods, the system works as a recuperated MGT. Both configurations exhibit nearly identical energy balance states, with approximately 60 % of the fuel energy being recovered through recuperation. The next frequent range lies between 800 and 900 W/m^2^, with over 600 h per year, signifying peak solar energy availability. In configuration 1, solar energy contribution, energy consumed in combustion and energy recovered in the recuperator are 7.9 (21 %), 15.1 (39 %) and 15.4 (40 %) MWh, respectively. These figures stand at 13.7 (32 %), 17.1 (47 %) and 6.9 (21 %) for configuration 2.

In configuration 1, the trend of energy balance aligns with the frequency hours until reaching DNI values of 800 W/m^2^. This implies a consistent distribution of energy sources up to this point. The majority of the energy is supplied by combustion, and recuperation has the second share. As DNI increases, the contribution of solar energy and recuperation grows, and eventually the recovery surpasses the combustion energy (Fig. 16a). As shown in Fig. 16 b, the trend differs in configuration 2. Recovered energy accounts for 60 %–40 % of combustion energy with lower values at higher DNI levels. Solar energy surpasses the share of recovered energy at DNI levels exceeding 600 W/m^2^.

The performance of these systems can be compared using different definitions of efficiency [54]. One such definition is fuel-to-power efficiency, calculated by dividing the generated power by the energy content of the consumed fuel, as shown in equation (19).

19 $$\eta_{fuel-power}=\frac{{\dot{W}}_{system}}{Q_{in,fuel}}$$

where $Q_{in,fuel}={\dot{m}}_{fuel}\times {LHV}_{fuel}$ represent the energy content of the consumed fuel in which ${\dot{W}}_{system}$ and ${LHV}_{fuel}$ denote the shaft power output and lower heating value of the fuel, respectively. Assuming that no water vapor condensation occurs downstream, the lower heat value is used rather than higher value [55].

Two other definitions of efficiency, “thermal-power” and “thermal-CHP”, define the amount of power and combined heat and power outputs from a unit amount of thermal input (considering all thermal sources) can be represented by equations 20, 21).

20 $$\eta_{thermal-power}=\frac{{\dot{W}}_{system}}{Q_{in,fuel}+Q_{in,Solar}}$$

21 $$\eta_{thermal-CHP}=\frac{{\dot{W}}_{system}+{\dot{Q}}_{CHP}}{Q_{in,fuel}+Q_{in,Solar}}$$

where ${\dot{Q}}_{CHP}$ represents the heat available downstream of the turbine exhaust and $Q_{in,Solar}$ denotes the total incident solar heat on the solar dish, which is defined in Equation (1). Solar contribution is considered as the amount of total incident solar heat on the solar dish divided by the total heat input of the system as $Q_{in,Solar}/\left(Q_{in,Solar}+Q_{in,fuel}\right)$.

**Fig. 17:**
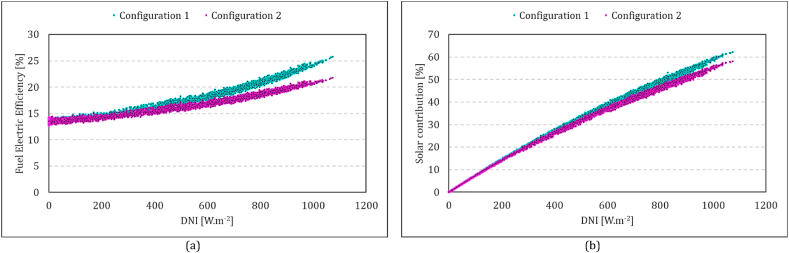
Comparison of the performance of both configurations in different DNI values: (a) Fuel power efficiency, (b) Solar contribution. Configuration (1) compressor flow recuperation, Configuration 2: receiver flow recuperation.

The fuel-power efficiency and solar contribution against DNI values for both configurations are compared in Fig. 17. The figure shows that as DNI increases, the contribution of the solar energy input slightly rises for Configuration 1 (Fig. 17b). Since the heat available at the solar dish is the same, it means less fuel consumption in Configuration 1 which results in higher fuel-to-power efficiency, as seen in Fig. 17 a. The maximum fuel-to-power efficiency in Configuration 2 is 22 %, whereas Configuration 1 achieves 26 %.

**Fig. 18:**
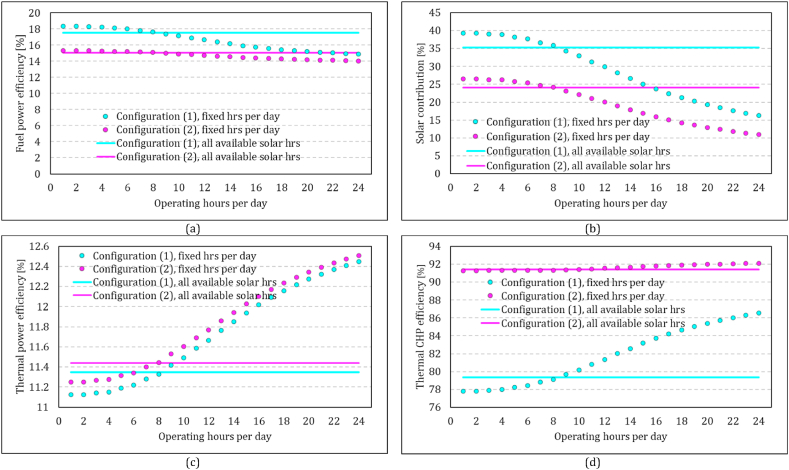
Comparison of the annual performance parameter of two configurations for different operating hour scenarios. (a) Fuel power efficiency, (b) Solar contribution, (c) Thermal power efficiency, (d) Thermal CHP efficiency Configuration (1) compressor flow recuperation (1), Configuration 2: receiver flow recuperation.

The results presented in this paper thus far have been derived from systems operating continuously 24 h a day, irrespective of the availability of solar energy. Alternatively, the simulation platform can be employed to predict the system performance at specific hours of the day or only when the solar energy is available. In Fig. 18, the overall performance parameter of the system is compared across both configurations for various operating hours. To achieve this, simulations were conducted for fixed hours of operation throughout all 365 days of the year. One-hour operation is assumed to start from 12:00 to 13:00, and the hours increased in both ends up to 24 h. This means 24 separate annual simulations for each configuration. Each dot plotted in this figure represents one of these simulation results. In another scenario, simulations were performed to estimate the system performance of day-time operations. It assumes system startup upon availability of direct irradiation and shutdown at the end of each day with termination of direct irradiation. In this scenario, the operating hours vary daily to align with solar availability. The continuous lines in this figure represents estimations based on this scenario. Fuel power efficiency, solar contribution, thermal power efficiency, and thermal CHP efficiency are displayed separately in this figure. Overall, configuration 1 outperforms configuration 2 in the first two parameters but lags behind in the latter two.

In the 24-h operation scenario, the majority of hours experience no solar energy, resulting in both configurations approaching the values of no solar MGT operation. Comparing based on operation hours offers a more precise assessment of the performance of each system under varying conditions. Fuel-power efficiency is shown in Fig. 18 a. In Configuration 1, the 24-h operational efficiency stands at 18 %, while in Configuration 2, it is 15 %. The annual value of this parameter in Configuration 2 range between 15 % and 14 %. In comparison, it can reach up to 18 % for 1-h operation at noon every day and remaining above 17 % for 12 h of operation. In the available solar operation scenario, it is 18 % and 15 % for Configuration 1 and Configuration 2, respectively. The higher fuel-power efficiency is somehow correlated with the solar contribution shown in Fig. 18 b. If the system operates only during hours with available solar energy, the share of the solar energy in the total energy input of the system is 35 % and 24 % for Configuration 1 and 2, respectively.

The thermal power efficiency of both configurations looks nearly identical in Fig. 18 c. This indicates that regardless of the daily operation hours, the total input energy for both systems remain the same for equivalent power outputs. However, the thermal CHP efficiency exhibits distinct trends (Fig. 18d). Configuration 2 has considerably higher values, around 91 %, for different operating hours scenarios. The values range between 78 % for 1-h and 87 % for 24-h operation in configuration 1. This suggests that Configuration 2 generates exhaust gas with a higher energy content available for extraction in a CHP cycle compared to Configuration 1, owing to the higher internal heat loss of the solar receiver in Configuration 1. The figure also implies that Configuration 2 could be more appropriate for the application with the constant demand for heat and power.

## 7 Conclusion and future work

To provide an accurate depiction of the operation of solar MGT systems, the annual performance of such systems was evaluated through an off-design simulation using real meteorological data. This simulation took into account the variation of ambient condition, DNI, and component off-design effects for both a conventional and an alternative configuration:

Configuration 1 compressor outlet recuperation.

Configuration 2 receiver outlet recuperation.

An off-design simulation platform was developed with the capability to estimate the effect of ambient temperature on the heat loss of the solar receiver and the overall performance of the system. The results of the off-design simulation were validated against experimental data obtained from a recuperated MGT system. Moreover, a limited number of experimental results from project “Solar Turbo CHP”, specifically for configuration 1 of a solar MGT system, were utilised to validate the accuracy of simulating the solar power within the integrated system. Both validation methods confirmed the credibility of the model in hybrid configurations.

The model was implemented for a test case Solar MGT system operation in Pretoria, utilising component characteristics of the “Solar Turbo CHP” project. Minute average values of the DNI and ambient condition were extracted for this location, and both configurations were analysed for the entirety the year 2022. The results were demonstrated for two representative days, Jun. 21, and Dec. 21, as high and low DNI days, respectively, to investigate the effects of changes in DNI and other BCs in greater details. The results of the entire year were presented, and the overall performance parameter of the system was compared.

The analysis revealed different behaviours between the two configurations at both subsystem and system performance levels. The solar receiver in Configuration 1 experiences considerably higher temperature compared to Configuration 2. The receiver heat loss, consequently, is much greater in Configuration 1, reaching a maximum value of 14.7 kW compared to 2.7 in Configuration 2, both at a DNI value of 1073.8 W/m^2^. The lower solar heat gain of Configuration 1 has a more substantial impact on fuel consumption meaning that under similar BC, Configuration 1 requires less fuel to generate the same amount of power. At high DNI values (∼1000 W/m^2^), the fuel power efficiencies are 26 % and 22 % for Configurations 1 and 2, respectively.

Since the availability of solar energy varies throughout the day, these configurations were analysed using different operating strategies. Fixed operating hours, ranging from 1 to 24 h per day, along with operation under all available solar conditions were considered, and overall system performance was compared. The comparison revealed that Configuration 1 outperformed Configuration 2 in terms of power fuel efficiency across all operating strategies. While the thermal power efficiency of the two systems is similar with a negligible difference, Configuration 2 demonstrated a 15 % higher CHP efficiency compared to Configuration 1 when operating under available solar scenario.

This study demonstrates that off-design simulation, coupled with a high-resolution distribution of BCs, can yield considerably different results compared to evaluation based solely on design point and average BC values. In this instance, the difference in the estimation of the fuel consumption for 365days×24housrs operation reached up to 26 %. This implies that a similar approach should be adopted for any hybrid system integrating a renewable energy source with a thermal engine.

This platform can be utilised to explore the effect of various components’ sizes and to design the system according to different demands. While the number of operating hours was discussed here in the operation strategy, the control strategy of “Constant TIT” was adopted for all simulations. The off-design model offers the capability to explore other control strategies for further investigations.

## Data availability statement

The supporting data of this study are available upon request from the corresponding author, S.H. The data are not publicly accessible as they contain information that could compromise the privacy of industrial participants.

## CRediT authorship contribution statement

**SeyedVahid Hosseini:** Writing – original draft, Validation, Methodology, Investigation, Formal analysis. **Yong Chen:** Writing – review & editing, Supervision, Project administration. **Hossein Madani:** Validation, Supervision, Project administration. **Mahmoud Chizari:** Writing – review & editing, Supervision.

## Declaration of competing interest

The authors declare the following financial interests/personal relationships which may be considered as potential competing interests:Seyedvahid Hosseini reports financial support was provided by 10.13039/501100000780European Commission Marie Sklodowska-Curie Actions. Hossein Madani reports financial support was provided by 10.13039/100014013UK Research and Innovation. If there are other authors, they declare that they have no known competing financial interests or personal relationships that could have appeared to influence the work reported in this paper.
